# Use of scenarios to explore conflict management practices of nurse unit managers in public hospitals

**DOI:** 10.4102/curationis.v42i1.1943

**Published:** 2019-06-27

**Authors:** Mabitja E. Moeta, Suzette M. du Rand

**Affiliations:** 1 Department of Nursing, University of Pretoria, Pretoria, South Africa; 2 Department of Nursing, Nelson Mandela University, Port Elizabeth, South Africa

**Keywords:** avoidance, conflict management, public hospital, nurse unit manager, workplace

## Abstract

**Background:**

Workplace conflict is common among nurses globally. Learning how to manage it may reduce related adverse consequences. Inappropriate management of conflict is attributed to decreased productivity, poor morale and financial loss for organisations. Nurse unit managers can play a key role by effectively managing workplace conflict in the units.

**Objectives:**

To explore how nurse unit managers manage conflict in public hospitals and subsequently to make recommendations on how to optimise conflict management skills of nurse unit managers.

**Method:**

A qualitative, explorative, descriptive and contextual study was conducted to explore how nurse unit managers managed conflict based on a scenario provided to them. Purposive sampling was used to select nurse unit managers working in three public hospitals. Eleven nurse unit managers participated in the study. Data were collected in two phases. In phase 1, a conflict scenario was developed in consultation with experienced nurse managers. The conflict scenario was used during phase 2, which involved individual face-to-face semi-structured interviews with nurse unit managers until data saturation. Tesch’s method of thematic synthesis was used to analyse the data. Literature review was undertaken to ascertain what is considered as an appropriate intervention in conflict management.

**Results:**

Three themes emanated from data analysis: nurse unit managers managed conflict appropriately, nurse unit managers avoided the conflict and nurse unit managers managed conflict inappropriately.

**Conclusion:**

While some of the nurse unit managers managed conflict appropriately, additional and continuous education and training is required to optimise the capacity and develop their conflict management competency. The findings could be integrated into orientation, training and preparation of nurse managers by health care organisations and educational institutions.

## Introduction and background

Workplace conflict is a global phenomenon occurring frequently in all settings where people work together. Literature reports the existence and prevalence of conflict among nurses in the workplace (Chartered Institute of Personnel and Development 2008:07; Higazee 2015:03). Although workplace conflict is not a contemporary issue, it continues to be a serious challenge for many organisations. The Center for Psychological Press (CPP) Global Human Capital Report commissioned in 2008 revealed that in the United States of America managers spend approximately $359 billion on resolving conflict, which is the same as 2.8 h per employee per week. Salem, Zakari and Al-Khamis (2009:218) also noted that the average nurse managers spend 20% to 50% of their work time resolving conflicts. It is not strange that health professionals frequently reporting conflict encounters (57%) in the workplace are nurses (Jerng et al. 2017:05). As such leading and managing nurses remains a challenging task for nurse managers.

Similarly, workplaces in South Africa are not immune to conflict situations. Armstrong, Rispel and Penn-Kekana (2015:04) revealed that nurse unit managers in South Africa spent about 11.5% of their work time in managing staff, including conflict resolution. Cunniff and Mostert (2012:09) discovered that 31.1% of South African employees experienced bullying in their workplaces, a typical symptom in conflict-ridden workplaces. In the workplace, bullying often emanates from the desire to control and insecurities from the manager when being challenged by subordinates. Managers who exhibit bullying behaviours tend to overlook interpersonal relations, resulting in strained relations (Cunniff & Mostert 2012:10). Other frequently reported sources of conflict include personality clashes, differences in methods of working and competition for resources within an institution (Chartered Institute of Personnel and Development 2008:02). Such relational behaviours if unmanaged can have undesired consequences for the individual and the organisation.

Individual effects related to conflict reported by nurses include psychosomatic symptoms, poor family life and increased incidents of grievances (Graham 2017:01; Nayeri & Negarandeh 2009:05). Some reports highlight the association between workplace conflict and sickness or absenteeism from work, escalating to physical attacks and, in extreme cases, depression and hospitalisation (Meier, Semmer & Gross 2014:39). Lower job satisfaction and staff turnover are common in conflict-ridden organisations and may render an organisation unattractive to potential employees. Based on serious effects conflict may have in an organisation, competent nurse managers are required to minimise the associated financial and reputational cost.

Reports indicate that, because of management failures, personnel working in public hospitals in South Africa experience extreme difficulties when attempting to deliver efficient health care services because of management failures. Such failures included the inability of line managers in successfully managing conflicts in the workplace (Fusheini, Eyes & Goudge 2017:75). Support from employers in workplaces where employees experience persistent conflict is essential. Mathumbu and Dodd (2013:87) have reported a positive correlation between job satisfaction and organisational support by managers. The organisational support from nurse managers implies that employers are fair and willing to create conducive working conditions, thereby minimising conflicts.

Nurse unit managers are identified as vital role players in conflict management because they represent the first level of management in a hospital and provide a link between the strategic vision of the organisation and frontline nurses (Cipriano 2011:01). Nurse unit managers also have a direct impact on human resource management and their ability to deal with conflict should be well developed. Globally, nursing management is considered a nursing speciality area wherein certain competencies are required of nurse unit managers to manage human and capital resources in the units. According to the American Organization of Nurse Executives (2015:06), conflict management is a competency requiring that those in supervisory roles undergo some form of preparation to be capacitated in facilitating interpersonal relations in their units.

In South Africa, the training and preparation of nurses on conflict management is presented at both undergraduate and postgraduate levels. Undergraduate nursing students undergoing training to become professional nurses are introduced to the concept of unit management, wherein a theoretical component on conflict management is included (Muller 2009:187). Often in nursing, student nurses progress through their studies to qualify as professional nurses, who are typically team leaders. These nurses have to manage relations between team members under their supervision, without having been practically evaluated on their ability to manage interpersonal relations. At postgraduate level, nurses are exposed to nursing management by enrolling for speciality programmes in either clinical or non-clinical postgraduate diploma or degree (Duma et al. 2012:07). A clinical programme is a specialist area in nursing science covering specific knowledge and skills required for nurses to perform in a particular field, such as intensive care, trauma nursing, childcare nursing and many others. Conversely, nurses may register with an accredited nursing education institution (NEI) for a non-clinical programme that focuses on specialised areas such as nursing education and management.

Postgraduate clinical programmes include subjects such as communication, team building, conflict management, etc. In the non-clinical programmes, Nursing Administration is a field of study where nurses specialising in management learn subjects such as strategic planning, financial management and human resource management in which conflict management is presented (Muller 2000:6). The assessment of competencies in conflict management is however mostly theoretical and based on the cognitive ability of students in written tests. The South African Nursing Council, in terms of the *Regulations for the Course for the Diploma in Nursing Administration* (Regulation 1501 of 1983 as amended by Regulation 2554 of 1885), prescribes that students registered for the Diploma in Nursing Administration spend at least 90 h in practical placement. Nurses who underwent such specialised training are somehow expected to manage conflict situations without difficulties (Armstrong et al. 2015:8). However, this is not always the case.

A challenge that South African public hospitals experience is that nurses often are promoted to managerial positions without relevant management qualifications. This poses serious challenges because certain competencies, such as conflict management, have to be acquired through formal training to facilitate understanding and application of principles in the workplace. According to Momberg (2011:96), organisational preparation to manage conflict has been associated with positive outcomes than managers who have not been formally trained. Therefore, it can be expected that managers who did not receive any formal training on conflict management, or those who do not continuously update their skills, may have difficulties in managing conflict situations.

Although some nurse managers in South African public hospitals do not have any formal qualifications, they receive some in-service education and training related to their human resource duties (Mokoka, Oosthuizen & Ehlers 2010:03). However, occasional training they receive requires regular update to maintain competency. Lack of training and upskilling are often found in managers who are uncertain and often unprepared to intervene when conflict arises in their units (Spagnol et al. 2010:796).

Managers approach conflict situations differently depending on a variety of factors, such as their personality, work experience, age and gender (Lahana et al. 2017:04). However, there is a paucity of literature in South Africa regarding conflict management by nurse managers. Strategies such as avoidance, competing, compromising, accommodating and collaboration have been identified in the literature. In conflict management, avoidance strategy is evident where parties in dispute ignore conflict or do not confront conflict directly (Booyens & Bezuidenhoudt 2014:374). Avoidance strategy is considered the least effective approach for conflict management (Moisoglou et al. 2014:80). Avoiding conflict may be appropriate when tensions have escalated and further discussion related to conflict is unproductive (Booyens & Bezuidenhoudt 2014:374). Competing strategy is an aggressive competitive strategy to attain personal goals at the cost of the other party. Alternatively, an accommodating strategy is a co-operative encounter where one party gives up their needs in order to preserve relationships. Muller (2009:187) describes compromising as a strategy whereby both parties attempt to find a common ground and sacrifice to keep peace. Lastly, collaborating is evident where conflict is dealt with in a confrontational manner through problem solving to satisfy the needs of both parties (Huber 2014:176). No single approach is more effective in all conflict situations and each may be useful under certain circumstances or context.

Ultimately, the lack of management capacity and approaches to manage conflict become an obstacle in an organisation and may impede the implementation of unit policies (Mokoka et al. 2010:02). To ensure operational efficiency and effective management of health care facilities, competent nurse managers are necessary to deal with workplace challenges. While not all conflict can be managed or requires the need to be managed, learning how to manage it can reduce associated undesirable outcomes.

## Problem statement

This study was based on observations of the researcher on incidences of workplace conflict in public hospitals. The researcher worked in public hospitals for a number of years and witnessed nurse unit managers avoiding confrontation of disgruntled nurses as a means of keeping peace in the workplace. On several occasions, nurse unit managers had difficulties resolving conflict in nursing units. As a result, high levels of conflict prevailed because of issues related to training, leave allocation and display of favouritism towards some nurses by nurse unit managers. This observed conduct resulted in hospital management introducing mechanisms including rotation system where nurses were allocated to different units on a monthly basis to avoid and minimise hostilities that existed among specific nurses. In some cases, nurse unit managers attempted to intervene in conflict situations, but they did so by threatening staff members with warnings of possible disciplinary actions or ‘shifting them to more labour-intensive units’. It became evident that nurse unit managers were either uncertain of or lacked the necessary skills to intervene when required. Uncertainty in such situations proved detrimental to peaceful operation and harmony in the units. Furthermore, a lack of concern and desire to intervene when conflict arose left staff aggrieved, demotivated and frustrated, resulting in high absenteeism and staff turnover. The reluctance by nurse unit managers alerted the researcher that there could be a lack of managerial competency regarding conflict management among nurse unit managers in public hospitals.

The researcher’s observations were supported in South African literature, where existence of poor management skills and abilities in public health facilities is widely reported (Cullinan 2016:01; Doherty 2014:23). A contributing factor to the deteriorating standard and quality of work life for employees has been linked to inadequate managerial skills among nurse managers, thus interfering with efficient operation of these health facilities. Nayeri and Negarandeh (2009:05) reported that managers who are autocratic, abuse power and do not support subordinates, contributed to conflict and subsequently to their inability to manage conflict. Spagnol et al. (2010:796) reveals that nurses are often unsure about how they need to intervene when faced with conflict situations. The above findings concur with the researcher’s observations in South African hospitals. Most of the international studies on conflict management are quantitative self-reported surveys and have not observed behaviours of nurses during conflict management (Kaimenyi 2014:58; Pandey, Sajjanapu & Sangwan 2015:77). Further, findings from self-reported studies using questionnaires are subjective and may lack authenticity, as it may not be a true reflection of competence (Demetriou, Ozer & Essau 2015:01). The researcher therefore identified the need to explore how nurse unit managers managed conflict in the public hospitals using a conflict scenario. This would allow the researcher to understand question responses from nurse unit managers based on the scenario provided.

## Aims and objectives

The aim and objectives of the study were the following:

- to explore the conflict management practices of nurse unit managers in the public hospitals
- to provide recommendations, based on the findings of the study, regarding how to optimise conflict management by nurse unit managers.

### Research question

The following research question guided the study: What are the conflict management practices of nurse unit managers in the public hospitals?

### Explanation of concepts

The following concepts are central to the discussion and will help readers understand the researcher’s interpretation of these concepts in the given context.

#### Conflict management

Conflict management involves designing effective strategies to moderate causes of conflict and, in so doing, improve methods to reduce conflict. It involves all measures implemented to reduce negative aspects and increase positive aspects of conflict (Huber 2014:174). In this study, the concept refers to the practice of preventing, identifying and resolving workplace differences in a rational and effective manner utilising managerial skills or established institutional platforms.

#### Nurse unit manager

The South African Nursing Council (SANC) defines a professional nurse as a person who is registered in a category under section 31(a) of the *Nursing Act* (Act 33 of 2005) in order to practise nursing or midwifery. Such a professional must have received education and training at a NEI accredited to provide a professional nurse education and training programme. In this study, the nurse unit manager is a professional nurse registered with SANC who holds a managerial position in a public hospital nursing unit. The person is responsible for day-to-day management and supervision of nursing personnel.

#### Staff nurse

Staff nurse is any person registered in terms of section 31(c) of the *Nursing Act* (Act 33 of 2005), who received education and training at a NEI accredited to provide staff nurse education and training programme. In this study, the staff nurse is a nurse employed in a public hospital, providing nursing care to patients of all ages. The staff nurse works under the supervision of a professional nurse within a nursing unit.

#### Auxiliary nurse

Auxiliary nurse is a person registered in terms of section 31 (1) (d) of the *Nursing Act* (Act 33 of 2005), who received education and training at a NEI that has been accredited to offer auxiliary nurse programme. In this study, auxiliary nurse is a nurse employed in a public hospital, providing basic nursing care to patients. This nurse works under the direct supervision of a professional nurse.

#### Public hospital

The term ‘public’ refers to a service provided to every citizen by the government and is financed by taxes. (Oxford Online Dictionary 2018). Public hospital in this study refers to general public sector, government-owned hospital providing comprehensive health care services to patients with different health needs.

### Contribution of the study to conflict management

The findings of the study have reaffirmed the findings of other studies concerning the impact of training on conflict management competency. The findings provide recommendations on how conflict management by nurse unit managers can be optimised within the context and constraints of a public sector hospital. The study also recommends the purposeful designing of conflict management training programmes by education institutions to equip managers with skills necessary for leadership challenges.

## Research methodology

### Research design

A qualitative, exploratory, descriptive and contextual study was used to enable the researcher to explore conflict management practices of nurse unit managers in public hospitals. According to Polit and Beck (2012:448), qualitative research is used when the nature and extent of the problem or phenomenon being investigated is not clear. Using qualitative research enabled a better understanding of how nurse unit managers managed conflict as related to the conflict scenario presented to them.

### Context of the study

The study was conducted in three public sector hospitals. Bed occupancy in the participating hospitals ranged from 450 to 570. Nursing units in which the nurse unit managers are based provide health care services for acute and chronic patients, with an average of 35 beds. Staffing in the units consists of three categories of nursing registered by the SANC: Professional Nurse, Staff Nurse and Auxiliary Nurse. Nurse unit managers included in the study were in charge of different units ranging from general medical surgical units, emergency care, maternity ward and operating theatre. The average number of nurses in each unit for both day and night shifts varied per unit and depended on the type of unit, but ranged from 16 to 40. The inclusion of the above units was influenced by the nurse unit manager’s willingness to participate in the study and they were not purposefully selected. Nursing units in participating hospitals operate on a 24-h basis. Nurse unit managers are the persons in charge of and supervising nurses in these units.

### Population and sampling

This study comprised nurse unit managers in three public hospitals. Each of the three participating hospitals has approximately eight nursing units, with one nurse unit manager in each unit. This equated to a total population of 24 unit managers available for this study. Purposive sampling was used to select nurse unit managers who met the criteria for participation. To be eligible for inclusion in the study, participants had to be employed as unit managers in a public hospital for at least 1 year at the time of the interview. Data saturation occurred after 11 interviews when no more new data were forthcoming. Table 1 indicates the number of nurse unit managers interviewed in each hospital.

**TABLE 1:** Number of nurse unit managers interviewed per hospital.

Hospital number	Number of nurse unit managers
1	4
2	4
3	3
**Total**	**11**

### Recruitment of participants

After obtaining permission from participating institutions, the researcher approached nursing service managers from these institutions and requested a list of the nurse unit managers employed in these hospitals who met the inclusion criteria. The researcher was invited to a meeting of the nurse unit managers where information about the study was shared and eligibility criteria were explained. A similar recruitment procedure for attending the nurse unit managers’ meetings was followed for all participating hospitals. Nursing service managers were given contact details of the researcher to allow those interested, even after the researcher had left, to contact the researcher should they require any clarity regarding the study or wish to participate. The researcher compiled a list of nurse unit managers who were willing to participate in the study with a schedule that had information regarding when nurse unit managers would be available for interview. After compiling the list, appointments for interviews were made with the participants. The same was done a day before the interview date.

### Trustworthiness

Trustworthiness is the degree of confidence researchers have in their research design, method, participants’ responses and context (Botma et al. 2010:182). Strategies of credibility, dependability, conformability and transferability were employed. A pilot study was conducted to validate conflict scenarios as well as to test the interview skills of the researcher. Rich data from participants were collected and verified by again listening to voice notes for ensuring confirmability. Member checking was done to ascertain if captured data were what participants meant. An independent coder was used and consensus was reached regarding themes that emanated from transcripts.

### Data collection method

Data collection took place in two phases. In phase 1, a conflict-related scenario that was to be used for data collection in phase 2 was developed. The researcher embarked on a literature search and engaged in informal conversations with nurses in clinical practice to ascertain which issues commonly breed conflict situations in a nursing unit setting. Input of experts from human resources, academics with qualifications in management and lastly nurse managers with experience in excess of five years was sought to verify the scenario. Experts were requested to indicate if the scenario was comprehensive, logical and practical given the circumstances within public hospital setting. It was important that the scenario was generic enough and relevant in any type of nursing unit, for example, surgical, medical and obstetric. Conflict scenario was about two professional nurses in disagreement about routine work issues such as study leave and on or off duties.

Phase 2 involved actual data collection that was done through face-to-face, semi-structured interviews. The researcher, who at the time of the study was a lecturer in nursing management at a university, conducted the interviews. The researcher facilitated conflict management workshops for nurses and was familiar with the processes involved and that made interviews less challenging. Because the researcher was not employed in the public hospitals, no conflict of interest existed. The interview had one central question and probing follow-up questions. A private room away from distractions in the hospital premises was used for interviews, which took approximately 45 min to complete. During the interviews, the printed conflict-related scenario was presented to participants. Then the participants were given time to read the scenario and clarify any misunderstandings, which took between 5 to 10 minutes. Once they indicated that they understood the scenario, they were asked to explain how they would manage the conflict in the scenario. The researcher probed participants until no new information emerged and stopped interviews when the participants indicated that there were no further inputs. Interviews were digitally recorded and field notes were taken by the researcher.

### Pilot study

Participants in the pilot study were sampled from the list obtained from the nursing service managers working at participating hospitals. Participants in the pilot study were from two of the three participating hospitals. Nurse unit managers who appeared on the list in alphabetical order were selected. Appointments were arranged to meet pilot nurse unit managers and obtain their consent for participation in the study. Data collection during the pilot study was audio recorded of which the participants were informed. A research supervisor was present and was subsequently provided with recordings. Then a discussion was held between the supervisor and the researcher to identify which interviewing skills were appropriate for the methodology adopted and also on how to improve the researcher’s interviewing skills. Privacy and confidentiality were maintained during data collection by ensuring that interviews took place in a private, noise-free area and that interruptions were minimised.

The conflict scenario was tested by giving it to the participants who were asked at the end of interviews about difficulties they experienced in understanding what was required of them. This was done to ensure that any possible errors were detected in the instructions, time limit and to identify unclear sections in the scenario. The research supervisor was given a copy of two pilot interviews as well as field notes for evaluation. A consensus between the researcher and the supervisor on themes of the pilot study was verified and the strength of instruments was confirmed. No major revisions were required for scenario or interview technique.

### Data analysis

Tesch’s (1990) method of thematic synthesis was used to analyse and code the data (Creswell, 2009:186). Data were transcribed verbatim and then coded by the researcher with assistance from an independent coder. The independent coder was provided with printed transcripts and voice recordings. Consensus was reached between the researcher and the independent coder regarding emerging themes. Table 2 highlights themes in terms of their classification.

**TABLE 2:** Classification of themes.

Themes	Sub-themes
1. Nurse unit managers managed the conflict appropriately	1.1 Nurse unit managers resolved the conflict between individuals
	1.2 Nurse unit managers managed the conflict appropriately in the units
2. Nurse unit managers avoided the conflict	
3. Nurse unit managers did not apply the accepted process to manage the conflict	3.1 Nurse unit managers did not resolve the conflict between individuals in an appropriate manner
	3.2 Nurse unit managers did not manage conflict in the unit in an appropriate manner

### Ethical considerations

Ethical clearance was obtained from Nelson Mandela University, Eastern Cape Provincial Health Research Committee (EC/2015RP34/229) and chief executive officers of respective hospitals. Permission was sought from all the participants. Ethical principles that were considered in this study include autonomy, beneficence and justice.

## Findings

Eleven nurse unit managers participated in the study. The majority of participants were women (*n* = 10, 91%), with the average age of participants being 52 years. Work experience of the participants varied from 6 to 15 years of working as nurse unit managers in public hospitals. There were seven participants with a postgraduate qualification in nursing management (64%). Table 3 highlights the demographic profiles of the participants.

**TABLE 3:** Demographic profiles of the participants.

Participant number	Gender	Age (years)	Postgraduate qualification in nursing management
01	Female	53	None
02	Female	51	Yes
03	Female	57	Yes
04	Female	56	Yes
05	Male	49	None
06	Female	54	None
07	Female	56	Yes
08	Female	53	Yes
09	Female	49	Yes
10	Female	54	None
11	Female	37	Yes

Data from interviews revealed three main themes: nurse unit managers managed conflict in an appropriate manner, nurse unit managers avoided the conflict and nurse unit managers did not apply the accepted process to manage conflict. A literature review was conducted to verify what is regarded as appropriate or inappropriate intervention in a workplace conflict.

### Theme 1: Nurse unit managers managed the conflict appropriately

The findings indicated that some participants managed conflict appropriately as it related to the scenario given, between individuals and generally in the unit. Appropriate management of conflict as used in this theme refers to all responses from participants that are likely to contribute to the effective resolution and management of conflict.

#### Subtheme 1.1: Nurse unit managers resolved the conflict between individuals

Most of the participants’ immediate response to the conflict was to diffuse the conflict situation. Nurse unit managers said that conflict should be resolved as soon as it becomes apparent. Participants also indicated that they would inform nurses on the code of conduct and explain expectations of the organisation. Some participants indicated that as soon as they become aware of a commotion in their unit as stated in the conflict scenario, they would approach the nurses and summon them to their office. Some participants’ intervention strategies to resolve conflict between individuals, which were regarded as appropriate, included not allowing the conflict to continue in public by providing privacy for nurses to resolve the conflict, being impartial and ensuring mutual respect during confrontation.

‘I must be just neutral and I must not let them speak at the same time; I must give them chances and make sure there is no dialogue between them, they must talk via me.’ (Participant 2)

A number of participants acknowledged that a safe environment was important in conflict management. A conducive environment to conflict resolution was described by participants as one in which nurses feel free to express their views without fearing that issues being discussed will be held against them. The response of one nurse unit manager after being aware of the conflict situation is as follows:

‘I believe that in any, in any conflict, there are two sides of the story. So I will call both in and get their sides of the story, from both of them at the same time.’ (Participant 5)

Some participants preferred an approach to conflict resolution where both nurses participate in the discussion. Most of the participants indicated that they would give both nurses a fair and equal chance to state their case. One participant stated that she would ensure that nurses give each other a chance to speak and raise their concerns. Most of the questions participants asked nurses in conflict were based on gaining a better understanding of what the problem is and why they engaged in the conflict in front of patients or in public.

‘I will ask them why they are arguing in front of patients. And then I will check what they are arguing about so that we can get to the bottom of the problem because I believe people cannot just argue without a reason.’ (Participant 7)

Nurse unit managers stated that patient care must take priority. Some participants indicated that they would encourage nurses to discuss issues among themselves and agree that what happened was wrong before nurse unit manager can intervene. Nurse unit managers also emphasised that nurses have to compromise and accommodate each other for the sake of patient care.

‘I will encourage this two to work along together, accommodating each other looking at the needs, at the same time we need to prioritise our core business. I mean we need to prioritise their needs, the core business which is nursing care.’ (Participant 11)

Nurse unit managers under this theme demonstrated skills as their interventions between individuals were deemed appropriate for the provided conflict scenario. Responses of nurse unit managers indicated that they are aware of behaviors necessary for effective conflict resolution between individuals.

#### Subtheme 1.2: Nurse unit managers managed the conflict appropriately in the units

The appropriate management of conflict in this context refers to all the management processes, structures and strategies employed by nurse unit managers in nursing units. Most of the participants were aware of the institutional platforms and stated that they will use management processes, such as disciplinary action, to reduce the impact of conflict and to ensure fairness in their management of conflict situations in the unit.

‘If I heard a noise it means that they were both fighting, so they must sign a warning for fighting in the unit number one, and then I must put that in their files but also I must notify the HR.’ (Participant 3)

Other participants focused on managing conflict in the unit by setting up systems and structures to moderate the impact of conflict. Some used policies and procedures of the institution as the basis for managing conflict. Other participants said they assume that all the nurses have gone through some form of orientation and have some knowledge about how issues are dealt with in the institution. One of the participants indicated that reliance on policies helps her manage conflict situations.

‘I am going to be straight to them. No crooked ways, this is how things are done and we work strictly according to the policies and procedures.’ (Participant 8)

It became clear that some nurse unit managers preferred to create a work environment in which conflict could be resolved promptly and constructively.

### Theme 2: Nurse unit managers avoided the conflict

Avoidance of conflict in this theme refers to deliberate mechanisms used to escape confronting conflict by nurse unit managers. Although avoiding conflict can be suitable at times, in this scenario it would not have been productive and helpful to avoid the conflict. Some participants avoided the conflict by accepting incorrect and unwarranted demands of nurses.

‘I don’t think there’s anyone who is wrong because that young nurse also has rights to go to school. We must just accept that conflict is part of growing up.’ (Participant 6)

Some nurse unit managers said that the resolution of conflict was not just the responsibility of line managers. It appeared as if the participants were avoiding the conflict by disowning it. When asked how they would deal with conflict, one participant said that she would refer nurses to various institutional platforms such as senior management or employee assistance programmes (EAPs) before exploring underlying causes and circumstances that lead to conflict.

‘I’ll get my Area Manager to come and join us. So that I can have a witness when I talk to both of them.’ (Participant 9)

‘I will take it further by taking the young one, let’s say I can refer her to the EAP you know. Employee’s practitioner [*general practitioner*], maybe there is a problem somewhere somehow.’ (Participant 9)

Some participants preferred harmony in the nursing unit as opposed to confronting the concerns raised. This behaviour symbolises a person avoiding the responsibility of managing a conflict, maybe in order to avoid tensions or maintain relationships in the unit rather than confront the problem. One participant said:

‘What I would do, what I am trying to achieve, I am trying to achieve team work; I am trying to achieve harmony. So I will accommodate them so that patient care cannot be compromised.’ (Participant 1)

In this theme, it became apparent that some nurse unit managers did not conceive the conflict presented to them in the scenario, as their immediate responsibility. In a conflict situation this can prove detrimental because employees may no longer trust the capabilities of their superior in future should there be grievances.

### Theme 3: Nurse unit managers did not apply the accepted process to manage conflict

Some nurse unit managers appeared to have difficulties dealing with conflict either between individuals or generally in the unit and intervened inappropriately. An inappropriate intervention refers to responses from nurse unit managers that are considered as ineffective in workplace conflict management. The timing when an intervention was effected in the conflict management process was taken into consideration to classify some responses as inappropriate.

#### Subtheme 3.1: Nurse unit managers did not resolve the conflict between individuals in an appropriate manner

Some participants intervened by suggesting solutions without exploring the root cause of conflict. This means that a thorough interrogation of the argument between the two nurses in conflict was not done.

‘I’ll just tell the younger one, you are wrong. First of all, who told you that you are going to the in-service training? So I’ll tell this one, don’t go. This one arranged and is going that’s all.’ (Participant 3)

Some participants stated that they would have the final say in the decision-making and on how to address or finalise the issue. The dictatorial approach to conflict was evident in the responses where participants said that there is no need to debate because it was evident who is wrong in this particular conflict situation.

‘I have to tell them same time; I don’t have to separate them according to this scenario. I’ll just tell the younger one, you are wrong.’ (Participant 4)

Most of the participants did not assess the state of affairs between nurses after conflict resolution. When participants were probed on what they would do after resolution, only a few said they will keep record of the incidents as well as monitor the efficacy of plan of action agreed upon.

‘Well… the process will have to be monitored closely and see if there will be no ill feelings towards each other, especially from the young nurse to see if she would not be bitter afterwards.’ (Participant 10)

The lack of confidence and interest in resolving conflict between employees, as seen under this theme, created an impression that nurse unit managers lacked appreciation of the delicate nature of a workplace conflict situation. Nurse unit managers must display confidence, demonstrate calmness, and be unbiased. However, for some, this was not the case in responding to the conflict scenario.

#### Subtheme 3.2: Nurse unit managers did not manage conflict in the unit in an appropriate manner

This subtheme highlights interventions and processes that participants said they would follow, which were unlikely to result in the resolution of conflict. Some participants did not regard conflict between two nurses as an issue that should be kept private. Participants did not ensure confidentiality because some indicated that they would also discuss the issue with the rest of staff during meetings in the unit.

‘I have to alert the other ones also because every morning we’ve got reviews in the morning every day. I have to also raise to other people to make them alert and correct that thing that happened yesterday.’ (Participant 2)

Participants referred the conflict to various institutional platforms such EAPs, labour unions and senior management. In this context, participants who chose to refer the conflict situation to such platforms did so prematurely, without having tried to intervene thereby deferring the responsibilities to a third party. This finding may suggest that participants did not want to be involved in conflict management until the end of the process. The point during the conflict resolution process that some participants stated that they would refer the matter is questionable. One participant said:

‘There are different labour unions in my institution. [*I will call them in*] so that we can sit down and try to solve the problem. They are also requested to bring own representatives as we discuss this problem.’ (Participant 9)

Nurse unit managers with postgraduate qualifications in management intervened appropriately in the conflict situation as compared to those with accumulated experience as nurse unit managers. Some nurse unit managers without management qualifications indicated that they lacked relevant skills and knowledge to manage conflict on the scenario given to them. Lack of competency was supported by the fact that most of the nurse unit managers highlighted the importance of training in order to improve their competencies pertaining to conflict management. Most of the participants were nevertheless eager to share how they managed other conflicts in their units as compared to how they would manage a conflict in the scenario provided.

## Discussion of findings

Throughout the study, it emerged that some participants could identify their responsibilities in conflict situation. Nurse unit managers were aware of conducive social and psychological environment required for conflict resolution. According to Johansen (2012:53), a non-punitive environment based on trust and openness contributes to effective conflict resolution among staff. Creating time and space for employees to express their feelings and concerns during a conflict can often help to clear the air and limits the possibilities of embarrassment, disruption of nursing care and potential physical violence (Advisory, Conciliation and Arbitration Service 2009:11). Consequently, giving people time to express feelings encourages learning and removes frustrations, resulting in trust and cooperation between team members.

Effective resolution of workplace conflict requires prompt intervention from the line manager (Cherry & Jacobs 2014:343). Nurse unit managers believed that conflict should be resolved as soon as it becomes apparent. Addressing conflict requires immediate intervention before the conflict escalates to intolerable levels. The CPP Global Human Capital Report (2008:11) revealed that 54% of participants in the study believed that for managers to resolve conflict effectively, they should identify and address underlying tensions timely before the situation deteriorates.

Removing involved parties from an audience is necessary to reduce the risk of conflict escalating or spreading to the entire unit. According to Heathfield (2016:01), when intervening in a conflict situation, managers should not meet parties separately, as this may interfere with objectivity and create a win–lose approach to conflict resolution. Meeting separately may breed mistrust and suspicions of bias or favouritism among staff members. Calling both parties in at the same time may heighten or moderate the tensions. Accurate assessment of circumstances by nurse unit manager is thus necessary before action.

Nurse unit managers perceived their role as a mediator who should be impartial and neutral. Impartiality requires fair and equal participation of both nurses to state their case in the discussion. Spagnol et al. (2010:796) discovered similar findings and reported that nurses regarded their role as that of a mediator when faced with workplace conflict. According to Doucet, Poitras and Chenevert (2009:344), neutrality in a conflict situation is more likely to have satisfactory outcomes and findings in de-escalation of conflicts. On the contrary, Van Gramberg et al. (2017:08) stated that taking a neutral position in conflict resolution has short-term benefits, whereas fairness and a just resolution process have long-term benefits.

Parties involved in conflict must be willing to compromise to some extent so that both parties are content with the outcome. Some nurse unit managers emphasised that nurses have to accommodate each other for the sake of patient care. The compromising approach suggested by participants is in line with the findings of Liu, Fu and Liu (2009:246), who in their study discovered that this approach is effective in reducing the negative effect of relationship conflict. Iglesias and De Bengoa Vallejo (2012:76) reported that compromising (27.7%) was the most frequently used approach to conflict resolution by nurses in their study.

Workplace policies regarding processes such as lines of communications and in-service training should be communicated with employees. Nurse unit managers in the current study also suggested that orientation of new employees should encompass relevant workplace policies. According to Thomas and Kilmann (2008:20), communicating available workplace procedures and policies to employees may contribute to effective conflict management. Availability of policies plays a pivotal role in the management of conflict as this occasionally provides managers with the basis for a fair decision-making process.

Overreliance on policies and processes by nurse unit managers as the basis for managing conflict may be ineffective and constitutes a competing style. Typically, competing style in conflict is evident when policies and rules are used to adjudicate on a matter and usually results in the satisfaction of one party’s needs at the expense of the other’s. A competing style has been reported to be ineffective as it causes intimidation and interferes with communication, discussion of alternative ideas and attempts at problem solving. In fact, competing style was found to be the least used strategy by nurses in a study by Baddar, Salem and Villagracia (2016:96).

Nurse unit managers avoided confronting the issues involved in resolving the conflict and seemed more concerned about maintaining relationships than resolving the problems. Possibly, they did not want to create the impression that they are too strict or they did not have the confidence to deal with conflict properly. Avoidance strategy is considered the least effective approach for conflict management as it creates a lose–lose approach for both parties. Remarkably, using avoidance strategy is common among nurse professionals. Moisoglou et al. (2014:80) reported that about 62% of nurses in their study used avoidance strategy for conflict resolution. Obied and Sayed Ahmed (2016:44) also reported that one-third of nurses in their study used avoidance style. Use of avoidance strategy was further attributed to a lack of experience, low self-confidence and mistrust in their professional abilities. However, Milton (2014:77) reported no relationship between the use of avoidance and inexperience. According to El Dahshan and Keshk (2014:138), managers who use avoidance strategy to resolve conflict do so to maintain relationships and curb turnover.

Alternatively, some participants appeared to have difficulty intervening in the conflict. Most nurse unit managers were challenged by logic in terms of which issues needed to be dealt with first and became haphazard in their interventions. When logic and process of conflict management are disregarded, findings are that authority is overlooked and opinions rather than factual policy statements may become standard practice of management. Ultimately, leading managers have to use intimidation and threats to avoid facing nurses. Use of intimidation by managers to manage difficult conflict situations at work was reported by Spagnol et al. (2010:797). Intimidation and threats may directly distort facts, resulting in prolonged conflicts. The use of intimidation and threats also encourages nurse unit managers to hide behind policy or shy away from confronting real issues.

Nurse unit managers referred nurses to various platforms prematurely for further interventions. The referral was done without proper exploration of the issues involved. Diagnosing and resolving conflicts require a clear understanding of differences and concerns of parties involved (Booyens 2010:542). When confronting or dealing with issues in workplace conflict, the scope and extent of the problem should be determined (Muller 2009:187). Furthermore, managing conflict requires the manager to explore if there are other parties involved or if the current conflict is the first encounter. If this is not done, disruptive team members may not be identified and remedial actions may not address the source of problems.

Nurse unit managers should take the final decision on the outcome of the conflict resolution process. This may lead to nurses disowning the agreement. Coming up with a conducive and favourable solution requires engagement through dialogue and negotiation to enhance trust among parties (Omisore & Rashidat Abiodun 2014:134). Kinnander (2011:42) reported that a fair process requires mutual agreement, wherein everyone is allowed to have his or her say in the process of conflict resolution and is involved in decision-making.

Nurse unit managers acknowledged that continuous training is required to improve conflict management competency. The most successful denominator for conflict resolution is formal training (Thomas & Kilmann 2008:02). Daud et al. (2013:132) reported a significant improvement in behaviour, confidence and skills for handling conflict in the workplace following training on conflict management.

## Limitations of the study

This study did not consider age, gender, qualifications and work experience of participants, which may have influenced participants’ approach to conflict management. Future studies should deliberate on such variables. The responses of participants were based on their understanding of the scenario and may not necessarily reflect their competence in conflict management in other contexts.

## Recommendations

This study aimed at exploring how nurse unit managers manage conflict in nursing units. It is evident that specialised training on conflict management should form part of all postgraduate courses for nurses, especially those involved in supervising subordinates in nursing units. For instance, clinical team leaders and nurse managers are expected to be role models for other staff members, and therefore need to actively resolve conflict in the workplace. Multi-disciplinary and inter-professional education should be part of all nurse managers’ training programmes in which human resource personnel participate. Such education will guarantee that nurse managers have the necessary knowledge and skills to deal with conflict. Further research is recommended on exploring how different variables such as age, gender and work experience of the nurse manager influence conflict management approaches.

## Conclusions

Conflict is an inherent and inevitable occurrence in all workplace settings. With the high costs and consequences unresolved conflict has on provision of nursing care, it is imperative that policymakers acknowledge the existence of workplace conflict and invest in resources to mitigate the impact. This study confirmed that while some nurse unit managers managed conflict appropriately, others still lacked capacity to do so and required additional training to optimise their conflict management competency.
